# Dietary inflammatory potential and risk of sarcopenia: data from national health and nutrition examination surveys

**DOI:** 10.18632/aging.202141

**Published:** 2020-12-14

**Authors:** Jiwen Geng, Linghui Deng, Shi Qiu, Haiyang Bian, Boyu Cai, Kun Jin, Xiaonan Zheng, Jiakun Li, Xinyang Liao, Yupei Li, Jiameng Li, Zheng Qin, Zhiwei Cao, Yige Bao, Baihai Su

**Affiliations:** 1 Department of Nephrology, and National Clinical Research Center for Geriatrics, West China Hospital of Sichuan University, Chengdu, Sichuan Province, China; 2 National Clinical Research Center of Geriatrics, The Center of Gerontology and Geriatrics, West China Hospital, Sichuan University, Chengdu, Sichuan, China; 3 Department of Gerontology, West China Hospital of Sichuan University, Chengdu, Sichuan Province, China; 4 Department of Urology, Institute of Urology, West China Hospital of Sichuan University, Chengdu, Sichuan Province, China; 5 Institute of Reproductive and Child Health and Department of Epidemiology and Biostatistics, Peking University School of Public Health, Beijing, China; 6 Institute for Disaster Management and Reconstruction, Sichuan University, Chengdu Sichuan Province, China; 7 Sichuan University West China College of Stomatology, Sichuan University, Chengdu, Sichuan Province, China

**Keywords:** inflammatory diet, dietary inflammatory index, sarcopenia, low lean mass, appendicular lean mass

## Abstract

This study used National Health and Nutrition Examination Surveys data from 1999 to 2006 to investigate the association between dietary inflammatory potential, represented by dietary inflammatory index (DII) scores, and the risk of sarcopenia in U.S. adults. A total of 25,781 participants were included in the study. The DII scores were calculated based on dietary information collected from 24-hour recalls. Men and women were classified as sarcopenic if appendicular lean mass (ALM) adjusted for BMI (ALM_BMI_) was <0.789 or <0.512, respectively. The covariates included comorbidities, dietary data, demographic data, and physical examination data. In a full-adjusted model, each unit of increase in DII score was associated with a 12% increase in risk of sarcopenia. When categorizing sarcopenia into tertiles, the adjusted effect size (relative to Tertile1) was 1.26 (95% CI, 1.07, 1.47) for Tertile 2 and 1.55 (95% CI, 1.31, 1.83) for Tertile 3. The trend test showed that the risk of sarcopenia increased with increasing DII tertiles, (P <0.0001). These findings demonstrate that dietary inflammatory potential correlates positively with the risk of sarcopenia and suggest that making ones diet inflammatory may reduce the incidence of sarcopenia and its associated negative health outcomes.

## INTRODUCTION

Inflammation is the body’s defensive response to tissue injury or inflammatory stimulants. However, if inflammation persists for a long time, it will trigger excessive production of pro-inflammatory cytokines, resulting in chronic systemic inflammation [1]. Many dietary factors are associated with inflammation. High sugar foods, refined grains, red and processed meats, and fried foods, are all thought as pro-inflammatory foods, which can increase the levels of inflammatory markers, such as tumor necrosis factor alpha (TNF-α), interleukin-6 (IL-6) and C-reactive protein (CRP) [2, 3]. In contrast, fish, fruits, legumes, nuts, olive oil, vegetables, and whole grains can reduce chronic inflammation [2]. For a more standardized assessment of the impact of diet on inflammation, the Dietary Inflammatory Index (DII^®^) was developed based on 1943 articles published from 1950 to 2010, from 11 countries. It reported the inflammatory effect of 45 dietary parameters, including flavonoids, food spices, macronutrients, and micronutrients, and each parameter was labeled with an inflammatory effect score. The total DII score positively correlates with the levels of inflammatory markers: the higher the score, the greater the dietary inflammatory potential [4].

Sarcopenia is the loss of functional strength and skeletal muscle mass resulting from advanced malnutrition, aging, disease, inactivity, or cachexia [5]. The Foundation for the National Institutes of Health (FNIH) proposed the definition of low lean mass (LLM) in 2014, an indicator of muscle mass based on imaging. The FNIH also suggested that LLM incorporates not only muscle mass, but also strength and function, to define “sarcopenia” [6]. Sarcopenia is a relatively common condition; the FNIH-reported prevalence is 20% in men, and 16% in women [7]. Sarcopenia is associated with negative health outcomes, including physical frailty, falls, disability [8], prolonged hospital stays, increased hospital costs [7, 9], and even an increased risk of all-cause mortality [10].

Chronic inflammation is one of the risk factors for the development and progression of cardiovascular disease, cancer, metabolic syndrome, and many other diseases [2, 11, 12]. Sarcopenia is also associated with inflammation, and with the increased levels of inflammatory markers CRP and IL-6 [13, 14]. Although some studies have suggested a correlation between diet, inflammation, and sarcopenia [15, 16], this link has not been conclusively demonstrated. Here, we used data representing the U.S. population to evaluate the dietary inflammatory potential for the risk of developing sarcopenia.

## RESULTS

### Baseline characteristics of participants

The sociodemographic characteristics and other covariates of the weighted distribution of included participants in accordance with the DII tertiles are shown in Table 1. The average age of the participants was 45.44 ± 12.22 years; 52.77% of them were males. The ranges of DII for tertiles 1-3 were -5.18 to 1.20, 1.20 to 2.92, and 2.92 to 5.71, respectively. Significant differences were observed for all included characteristics among the DII tertiles. Compared to Tertile 1 and Tertile 2, participants in Tertile 3 were younger, were often females, current smokers, and had a lower poverty to income ratio. The rate of sarcopenia using the FNIH ALM definition adjusted for BMI (ALM_BMI_) was 17.61%, which was much lower than the sarcopenia rate (28.47%) calculated using the FNIH ALM definition.

**Table 1:** Baseline characteristics of participants.

	**Overall**	**Tertile 1**	**Tertile 2**	**Tertile 3**	**P-value**
	**(n=25781)**	**(n=8578)**	**(n=8694)**	**(n=8509)**	
**DII**	1.84	-5.18 to 1.20	1.20 to 2.92	2.92 to 5.71	
**Mean**					
**Age, mean ± SD (years)**	45.44±12.22	48.72±11.47	44.75±12.35	42.85±12.42	<0.001
**Proportion (%)**					
**Sex**					<0.001
**Male**	52.77	63.78	52.80	41.64	
**Female**	47.23	36.22	47.20	58.36	
**Race**					<0.001
**Mexican American**	26.45	27.23	27.01	25.10	
**Other Hispanic**	4.05	4.00	4.20	3.94	
**Non-Hispanic White**	40.60	45.28	39.37	37.13	
**Non-Hispanic Black**	25.09	19.71	25.64	29.96	
**Other Race**	3.81	3.78	3.78	3.88	
**Ratio of family income to poverty**					<0.001
**<1.3**	33.02	28.69	33.07	37.32	
**1.3-3.5**	38.12	35.91	38.86	39.57	
**>3.5**	28.87	35.40	28.07	23.11	
**Education level**					<0.001
**Less than high school**	32.28	28.54	32.22	36.13	
**High school or General educational development (GED)**	24.06	22.06	23.86	26.29	
**Above high school**	43.66	49.40	43.93	37.58	
**Marital state**					<0.001
**Married or living with partner**	63.91	67.51	64.12	60.04	
**Living alone**	36.09	32.49	35.88	39.96	
**BMI**					<0.001
**<25**	47.24	45.49	47.54	48.70	
**≥25**	52.76	54.51	52.46	51.30	
**Comorbidity index**					<0.001
**0**	60.18	62.51	60.41	56.79	
**1**	29.73	28.41	29.27	32.02	
**≥2**	10.09	9.08	10.32	11.19	
**Smoking state**					<0.001
**Never**	50.92	51.06	51.91	49.62	
**Former**	29.93	33.12	28.70	27.08	
**Current**	19.14	15.82	19.39	23.30	
**Alcohol intake per week**					<0.001
**Never**	23.55	19.23	23.26	30.13	
**Up to once a week**	52.88	50.48	54.27	54.69	
**2-3 times a week**	12.30	14.37	12.17	9.44	
**4-6 times a week**	5.81	8.46	4.96	2.98	
**Daily or more**	5.47	7.45	5.34	2.75	
**Physical activity**					<0.001
**Less than moderate**	31.73	29.13	31.81	34.46	
**Moderate**	23.73	24.60	23.69	22.82	
**Vigorous**	44.55	46.27	44.50	42.72	
**Sarcopenia (ALM_BMI_)^1^**					<0.001
**No**	82.39	85.94	81.09	80.14	
**Yes**	17.61	14.06	18.91	19.86	
**Alternative Sarcopenia (ALM-only)^2^**					<0.001
**No**	71.53	78.26	70.16	66.15	
**Yes**	28.47	21.74	29.84	33.85	

In sensitivity analysis, dietary inflammatory index was converted from a continuous variable to a categorical variable (tertiles).

Note: Mean ± SD for continuous variables: P value was calculated by weighted linear regression model. Number (%) for Categorical variables: P value was calculated by weighted chi-square test.

^1^Sarcopenia: using the FNIH ALM adjusted for BMI(ALM_BMI_) definition.

^2^Alternative Sarcopenia: using the FNIH ALM-only definition.

### Association between dietary inflammatory index and sarcopenia

The association between dietary inflammatory index and sarcopenia is shown in Table 2. Model 1, an unadjusted model, indicated that sarcopenia positively correlated with DII scores. In Model 2, which adjusted for sociodemographic data (age, education level, marital status, poverty to income ratio, race, and sex) and self-reported history of diseases, the association between exposure variables and outcomes was still stable. In Model 3, which adjusted for all covariates, each unit of increased DII score was associated with 12% increased risk of sarcopenia.

**Table 2:** Association of dietary inflammatory index with sarcopenia.

**Dietary inflammatory index**	**β^1^ (95% CI^2^), P value**		
	**Model 1^3^**	**Model 2^4^**	**Model 3^5^**
	**(n=25781)**	**(n=11474)**	**(n=10653)**
**Sarcopenia (ALM_BMI_)^6^**			
**Continuous**	1.11 (1.09, 1.13) <0.0001	1.12 (1.08, 1.16) <0.0001	1.12 (1.08, 1.16) <0.0001
**Tertiles**			
**Tertile 1(-5.18 to 1.20)**	1.00 (reference)	1.00 (reference)	1.00 (reference)
**Tertile 2(1.20 to 2.92)**	1.43 (1.31, 1.55) <0.0001	1.27 (1.09, 1.47) 0.0018	1.26 (1.07, 1.47) 0.0043
**Tertile 3(2.92 to 5.71)**	1.51 (1.40, 1.64) <0.0001	1.58 (1.35, 1.85) <0.0001	1.55 (1.31, 1.83) <0.0001
**DII group trend**	1.12 (1.09, 1.14) <0.0001	1.13 (1.08, 1.17) <0.0001	1.12 (1.07, 1.17) <0.0001
**Alternative Sarcopenia (ALM-only)^7^**			
**Continuous**	1.16 (1.14, 1.18) <0.0001	1.13 (1.09, 1.17) <0.0001	1.12 (1.08, 1.16) <0.0001
**Tertiles**			
**T1(-5.18 to 1.20)**	1.00 (reference)	1.00 (reference)	1.00 (reference)
**T2(1.20 to 2.92)**	1.53 (1.43, 1.64) <0.0001	1.40 (1.20, 1.63) <0.0001	1.38 (1.17, 1.61) <0.0001
**T3(2.92 to 5.71)**	1.84 (1.72, 1.97) <0.0001	1.64 (1.40, 1.92) <0.0001	1.61 (1.36, 1.90) <0.0001
**DII group trend**	1.18 (1.15, 1.20) <0.0001	1.14 (1.10, 1.19) <0.0001	1.14 (1.09, 1.19) <0.0001

In sensitivity analysis, dietary inflammatory index was converted from a continuous variable to a categorical variable (tertiles).

^1^β: effect sizes;

^2^95% CI: 95% Confidence interval;

^3^Model 1: no covariates were adjusted;

^4^Model 2: adjusted for gender; age; race; ratio of family income to poverty; education level; BMI; comorbidity index; smoking; alcohol intake per week;

^5^Model 3: adjusted for gender; age; race; ratio of family income to poverty; education level; marital; BMI; comorbidity index; smoking; alcohol intake per week; physical activity;

^6^Sarcopenia: using the FNIH ALM adjusted for BMI(ALM_BMI_) definition;

^7^Alternative Sarcopenia: using the FNIH ALM-only definition.

**Figure 1:**
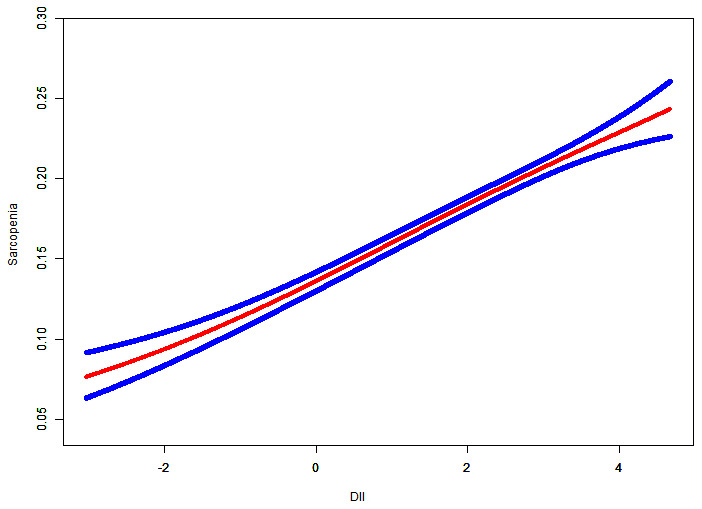
**Relationship between dietary inflammatory index and sarcopenia.** Risk of sarcopenia (red) with 95% CIs (blue) determined using the generalized additive model.

Penalized spline method and GAM model were used to evaluate the nonlinear relationship between dietary inflammatory index and sarcopenia. The result was negative; this meant that there was no nonlinear relationship between exposure variables and outcomes (Figure 1).

To perform sensitivity analysis, we converted dietary inflammatory index from a continuous variable to a categorical variable (tertiles). In terms of sarcopenia, the adjusted effect size (reference to Tertile 1) was 1.26 (95%CI, 1.07, 1.47) for Tertile 2, and 1.55 (95%CI, 1.31, 1.83) for Tertile 3. In addition, we assessed the association between dietary inflammatory potential and alternative sarcopenia. The results showed that each unit of the increased DII score was associated with 12% increased risk of alternative sarcopenia. When the dietary inflammatory index was converted into tertiles for alternative sarcopenia, the adjusted effect size (reference to Tertile 1) was 1.38 (95% CI, 1.17, 1.61) for Tertile 2, and 1.61 (95% CI, 1.36, 1.90) for Tertile 3. The trend test also showed that with the increase of DII tertiles groups, the risk of both sarcopenia and alternative sarcopenia increased (P for trend <0.0001).

### Subgroup analyses

We tested interactions with all covariates presented in Table 1; the results are shown in Table 3. Regarding the correlation between DII scores and sarcopenia, the test for interaction was significant for educational level (P for interaction = 0.0005). However, we did not detect any significant correlation between DII scores and alternative sarcopenia.

**Table 3:** Subgroup analysis.

**DII**	**Sample Size**	**Sarcopenia (ALM_BMI_)^1^**	**P interaction**	**Alternative Sarcopenia (ALM-only)^2^**	**P interaction**
	10653	β (95%CI), P value		β (95%CI), P value	
**Sex**			0.5027		0.1372
**male**	5895	1.11 (1.06, 1.16), <0.0001		1.16 (1.09, 1.24), <0.0001	
**female**	4758	1.14 (1.07, 1.21), <0.0001		1.10 (1.05, 1.15), <0.0001	
**Age**			0.5956		0.2833
**<65**	8527	1.11 (1.06, 1.16), <0.0001		1.10 (1.05, 1.14), <0.0001	
**≥65**	2126	1.13 (1.07, 1.20), <0.0001		1.14 (1.07, 1.22), <0.0001	
**Race**			0.1812		0.8442
**Mexican American**	2196	1.07 (1.01, 1.13), 0.0291		1.09 (1.02, 1.17), 0.0104	
**Other Hispanic**	415	1.19 (1.00, 1.40), 0.0477		1.20 (1.00, 1.45), 0.0560	
**Non-Hispanic White**	5689	1.17 (1.10, 1.23), <0.0001		1.13 (1.08, 1.19), <0.0001	
**Non-Hispanic Black**	2021	1.02 (0.87, 1.20), 0.7724		1.11 (0.96, 1.28), 0.1546	
**Other Race**	332	1.13 (0.90, 1.40), 0.2901		1.09 (0.92, 1.30), 0.2938	
**Ratio of family income to poverty**			0.2094		0.2440
**<1.3**	2549	1.08 (1.01, 1.16), 0.0196		1.11 (1.04, 1.20), 0.0029	
**1.3-3.5**	4104	1.11 (1.05, 1.17), 0.0006		1.09 (1.03, 1.15), 0.0044	
**>3.5**	4000	1.18 (1.10, 1.27), <0.0001		1.17 (1.10, 1.24), <0.0001	
**Education level**			0.0005		0.0753
**Less than high school**	2844	1.05 (0.99, 1.12), 0.0854		1.07 (1.00, 1.14), 0.0587	
**High school or General educational development (GED)**	2548	1.07 (0.99, 1.15), 0.0925		1.10 (1.02, 1.18), 0.0146	
**Above high school**	5261	1.23 (1.16, 1.32), <0.0001		1.17 (1.11, 1.23), <0.0001	
**Marital state**			0.6598		0.6575
**Married or living with partner**	7129	1.11 (1.06, 1.16), <0.0001		1.13 (1.08, 1.18), <0.0001	
**Living alone**	3524	1.13 (1.06, 1.21), 0.0004		1.11 (1.04, 1.18), 0.0008	
**BMI**			0.1705		0.4637
**<25**	3347	1.05 (0.96, 1.16), 0.3023		1.13 (1.08, 1.19), <0.0001	
**≥25**	7306	1.13 (1.08, 1.18), <0.0001		1.10 (1.04, 1.17), 0.0012	
**Comorbidity index**			0.3833		0.6410
**0**	6547	1.10 (1.05, 1.16), 0.0003		1.12 (1.07, 1.18), <0.0001	
**1**	3097	1.16 (1.09, 1.23), <0.0001		1.10 (1.03, 1.18), 0.0059	
**≥2**	1009	1.08 (0.98, 1.19), 0.0999		1.17 (1.04, 1.31), 0.0073	
**Smoking**			0.3647		0.9107
**never**	4885	1.14 (1.08, 1.21), <0.0001		1.11 (1.06, 1.17), <0.0001	
**former**	3526	1.12 (1.05, 1.18), 0.0002		1.13 (1.06, 1.21), 0.0002	
**current**	2242	1.06 (0.97, 1.16), 0.2266		1.12 (1.03, 1.22), 0.0069	
**Alcohol intake per week**			0.2969		0.5947
**never**	2407	1.15 (1.08, 1.24), <0.0001		1.11 (1.03, 1.19), 0.0065	
**Up to once a week**	5702	1.11 (1.05, 1.17), 0.0001		1.15 (1.09, 1.20), <0.0001	
**2-3 times a week**	1335	1.02 (0.90, 1.15), 0.7491		1.04 (0.94, 1.16), 0.4660	
**4-6 times a week**	655	1.07 (0.87, 1.30), 0.5267		1.11 (0.95, 1.29), 0.1980	
**Daily or more**	554	1.23 (1.05, 1.45), 0.0114		1.14 (0.96, 1.34), 0.1269	
**Physical activity**			0.1236		0.5547
**Less than moderate**	3997	1.08 (1.03, 1.14) 0.0027		1.14 (1.07, 1.21) <0.0001	
**Moderate**	3101	1.13 (1.05, 1.21) 0.0005		1.13 (1.06, 1.21) 0.0002	
**Vigorous**	3555	1.20 (1.10, 1.31) <0.0001		1.09 (1.02, 1.16) 0.0109	

The showing results of subgroup analysis was adjusted for all presented covariates except effect modifier.

^1^Sarcopenia: using the FNIH ALM adjusted for BMI(ALM_BMI_) definition.

^2^Alternative Sarcopenia: using the FNIH ALM-only definition.

Even though the prevalence of sarcopenia was reported to be increased among older populations [17, 18], we did not observe any significant dependence on age (P = 0.5956 for sarcopenia, P = 0.2833 for alternative sarcopenia). In addition, we did not find any significant dependence on physical activity (P = 0.1236 for sarcopenia, P = 0.5547 for alternative sarcopenia).

Furthermore, our results indicated that the correlation between DII scores and sarcopenia was similar in participants with different alcohol consumption, body max index, comorbidity index, marital status, sex, smoking status, race, and ratio of family income to poverty.

## DISCUSSION

Our results demonstrate that higher dietary inflammatory DII scores are associated with the increased risk of sarcopenia. The results were similar for both FNIH-defined sarcopenia types (adjusted for BMI, and ALM-only). In addition, the positive correlation between DII scores and the risk of sarcopenia was not affected in different subgroups. To our knowledge, this study is the first to show the association between dietary inflammatory potential and the risk of sarcopenia.

Sarcopenia has been associated with increased levels of myostatin, a negative regulator of muscle mass [19], and skeletal muscle inflammation [20]. Sarcopenia has been observed in patients with chronic inflammatory conditions [21], and anti-inflammatory therapy reduces inflammation-induced muscle weakness [22]. Previous studies have suggested that inflammation parameters inversely correlate with muscle strength [23, 24]. Particularly, sarcopenia has been associated with increased serum CRP levels [14]. Inflammation is a major biological process regulating the interaction between environment and organisms, and diet plays a crucial role in the environment [25]. Some types of food are thought as pro-inflammatory foods, including high sugar foods, refined grains, red and processed meats, and fried foods [2]. Long-term diets rich in these foods tend to increase chronic inflammation, which may lead to sarcopenia. Our results demonstrating the positive correlation between high DII scores and sarcopenia are consistent with most previous studies on inflammation and sarcopenia. However, a previous review suggested that age related decline in hormones, neurodegenerative processes, and disability, rather than inflammation, were associated with the development of sarcopenia [26]. Although the pathogenesis and mechanisms of sarcopenia are controversial, our findings provide a strong evidence for the effect of dietary inflammatory potential on sarcopenia.

In subgroup analysis, although the interaction test in educational level was statistically significant, its direction and trend were consistent with overall results. This might be caused by the bias caused by the insufficient sample size. Although age was thought to be a risk factor for sarcopenia, we did not find any significant interaction with age, suggesting that age was not a limiting factor in the positive association found between DII scores and the sarcopenia risk. Sarcopenia is defined as a loss of functional strength and skeletal muscle mass [5]. Even though physical activity was thought to be a protective factor against sarcopenia [27], we did not find any significant interaction with physical activity. The results suggest that inflammatory diet increases the risk of sarcopenia regardless of physical activity. Using univariate and multivariate analyses, we found a negative association between DII scores and physical activity (Supplementary Table 1). To elucidate the association between physical activity and sarcopenia, we also assessed the relationship between DII scores and muscle strength. Since the NHANES database from 1999 to 2006 did not contain any data on grip strength, we used the data of isokinetic strength of knee extensions (quadriceps) instead. The results suggested a negative association between the DII scores and muscle strength (Supplementary Table 2). Together, our analysis of a pooled sample representing both males and females, diversity of race, multiple geographic regions in the US, and a range of health and functional states, indicated the positive correlation between inflammatory diet and the risk of sarcopenia.

The positive association between DII scores and sarcopenia was observed in both types of FNIH-defined sarcopenia, sarcopenia defined by ALM adjusted for BMI (ALM_BMI_) and alternative sarcopenia defined by ALM-only. Of note, the risk of alternative sarcopenia was always higher than the risk of sarcopenia in the same situation, while the directions and trends in both were similar. The FNIH recommended that ALM_BMI_ should be used over ALM-only [6]. Body mass adjustment had noticeable effects especially in women, for it can evaluate individual’s weakness and muscle more accurately [28]. Therefore, our results indicate that sarcopenia defined by ALM_BMI_ has more reference value and clinical significance than alternative sarcopenia defined by ALM-only.

An important aspect of our study is that it analyzed a representative sample of U.S. population. All data in the NHANES were collected using standardized protocols that minimized any possible bias. In addition, to ensure that our results can be applied to a wide range of people, we considered many covariates including sociodemographic information, health, and functional states. By comparing two types of FNIH-defined sarcopenia, we were able to show their similarities and differences. However, the cross-sectional study design could not provide a sufficient evidence for temporal relations and causal inference. In addition, the dietary information was limited, because it was obtained from 24-hour recalls. This method of collecting data has a relatively large intra-person variability, which may lead to misclassification in categorizing the DII tertiles. Although the DII consisted of 45 food parameters, only 27 parameters were collected from 24-hour recalls due to the questionnaire setting. However, previous studies showed that the predictive ability was not affected when the DII score was calculated by only 27 or 28 food parameters [29, 30]. Furthermore, the obtained dietary information about the food consumed during a 24-hour period may not reflect the long-term diet habits.

Together, our results show that the dietary inflammatory potential, represented by high DII scores, positively correlates with the risk of sarcopenia, suggesting that decreasing the inflammatory diet might reduce the incidence of sarcopenia and its associated negative health outcomes.

## MATERIALS AND METHODS

### Data source and participants

The National Health and Nutrition Examination Surveys (NHANES), an ongoing repeated cross-sectional study administered by the Centers for Disease Control and Prevention (CDC), is a program designed to assess the health and nutritional status of population in the United States. The current NHANES, also known as Continuous NHANES, refers to the two-year cycles of data produced since 1999. All NHANES cycles performed similar operation procedures. Database in each cycle is divided into five sections: Demographics, Dietary, Examination, Laboratory, and Questionnaire. The survey examines a nationally representative sample of about over five thousand people each year across the U.S. The NHANES program was approved by the National Center for Health Statistics (NCHS) Ethics Review Board, and all participants have signed informed consent. All NHANES data and information are publicly available at https://www.cdc.gov/nchs/nhanes/index.htm. We performed analysis based on the data from four 2-year NHANES survey cycles: 1999-2000, 2001-2002, 2003-2004, and 2005-2006. We selected 25781 (1999–2000: 5607 cases; 2001–2002: 7186 cases; 2003-2004: 6846 cases; 2005-2006: 6142 cases) out of 41474 (1999–2000: 9965 cases; 2001–2002: 11039 cases; 2003-2004: 10122 cases; 2005-2006: 10348 cases) participants for the analysis. We excluded individuals with missing body composition measures (n=14445), missing single 24-hour dietary recall (24HR) data (n=1146), and missing data for covariate (n=103) (Supplementary Figure 1). The Institutional Review Board at the CDC provided the human subject approval for this study.

### Dietary inflammatory index measurement

We evaluated the baseline dietary intake by 24-hour dietary calls (24HR) that were validated by the Nutrition Methodology Working Group [31]. The 24-hour call data collected information about drinks and food consumed during the 24-hour period prior to the interview. Total intake of energy, nutrients, and non-nutrient foods was estimated, and detailed information about all foods and beverages was recoded in a standard 24-hour dietary interview format. We used the Dietary Inflammatory Index (DII^®^) to assess the impact of diet on inflammation and used the 24HR data to calculate the DII scores. The DII, developed in 2009 to measure the effect of diet-induced inflammation, consists of 45 food parameters [32]. 27 of these parameters were available from the 24HR data: alcohol, β-carotene, cholesterol, carbohydrates, energy, fats, fibers, folic acid, iron, magnesium, zinc, vitamin A, vitamin B-6, vitamin B-12, vitamin C, vitamin D, vitamin E, mono-unsaturated fatty acid, protein, niacin, riboflavin, (n-3) fatty acids, (n-6) fatty acids, poly-unsaturated fatty acids, saturated fat, selenium, and thiamin. Inflammatory effect scores for dietary components used for calculation of the DII are shown in Supplementary Table 3 [4]. Positive numbers represent pro-inflammatory effect, while negative numbers represent anti-inflammatory effect. The inflammatory effect scores were used to calculate an overall DII score. In previous studies, the DII scores calculated using only 27 or 28 food parameters did not influence the predictive ability [29, 30].

### Body composition measurement

Body composition measurement was assessed by dual energy x-ray absorptiometry (DEXA) QDR- 4500 Hologic Scanner (Bedford, MA, USA). The data of total skeletal muscle mass, appendicular lean mass (ALM), fat mass, and bone mineral content were collected. The NHANES also reported total body fat percent and lean mass percent. All tests were performed by trained technicians. All metal objects (except false teeth and hearing aids) were removed during the measurements. It is noted that the DXA scan had limits on height (maximum 192.5 cm) and weight (136.4 kg), and individuals outside this range were excluded.

ALM was defined as the sum of muscle mass of all four upper/lower extremity limbs. In terms of sarcopenia, we used the two definitions proposed by FNIH in 2014: ALM adjusted for BMI(ALM_BMI_) and ALM-only. Men were classified as sarcopenia if ALM_BMI_ <0.789, and women<0.512; men were classified as alternate sarcopenia if ALM <19.75 kg, and women<15.02kg.

### Covariates

For covariates, continuous variables included age (year), body mass index (BMI, kg/m^2^), comorbidity index, and ratio of family income to poverty. Information on comorbidities that constitute the Charlson comorbidity index (CCI), included acquired immunodeficiency syndrome, cerebrovascular disease, congestive heart failure, diabetes hemiplegia, diabetes with end organ damage, liver disease, lymphoma, moderate or severe renal disease, chronic pulmonary disease, any tumor, connective tissue disease, myocardial infarction, dementia, leukemia, peptic ulcer disease, and peripheral vascular disease [33].

Categorical variables included alcohol intake per week (never, up to once a week, 2-3 times a week, 4-6 times a week, daily or more), educational level (less than high school, high school or general educational development, above high school), marital status (Married or living with partner, Living alone), physical activity (less than moderate, moderate, vigorous), race (Mexican American, other Hispanic, non-Hispanic white, non-Hispanic black, other race), sex (male, female) and smoking status (never, former, current).

### Statistical analysis

All statistical analyses were conducted according to CDC guidelines (https://wwwn.cdc.gov/nchs/nhanes/tutorials/default.aspx). A sample weight was taken into consideration and assigned to each participant [34]. Marked variance was calculated, and proposed weighting methodology was used. Continuous variables were presented as mean ± standard deviation (SD). Categorical variables were presented as a frequency or as a percentage. Weighted linear regression model (for continuous variables) or weighted chi-square test (for categorical variables) were used to calculate the differences among different DII groups (tertiles). To explore the association between dietary inflammatory potential and sarcopenia, our statistical analyses included the following main steps.

Step 1: We employed weighted univariate and weighted multivariate line regression model. Three models were constructed and used in our analyses: model 1, no covariate was adjusted; model 2, sociodemographic data and self-reported history of diseases were adjusted; model 3, the covariates in model 2 and other covariates presented in Table 1 were adjusted.

Step 2: To address the nonlinearity of DII and sarcopenia, we conducted smooth curve fitting (penalized spline method) and weighted generalized additive model (GAM).

Step 3: Weighted stratified line regression models were used to perform subgroup analyses. All continuous covariables were converted into categorical variables according to their clinical cut points or tertiles, and used to perform an interaction test. We used interaction terms between subgroup indicators to test the effect modification in subgroup, followed by a likelihood ration test.

To ensure the robustness of data analysis, we conducted the following sensitivity analysis. First of all, we converted DII into a categorical variable by tertile and performed testing for linear trends. One purpose was to verify the results of DII as a continuous variable; another was to determine whether there was a nonlinear relationship. All steps described above were also performed to assess the association between dietary inflammatory potential and alternative sarcopenia.

All analyses were conducted using the Empower (R) (www.empowerstats.com; X&Y Solutions, Inc., Boston, MA, USA) and statistical package R (http://www.R-project.org, The R Foundation) with a significance threshold of 2-sided P < 0.05.

## Supplementary Material

Supplementary Figure 1

Supplementary Tables
