# Supplementary figures and images for: Dietary inflammatory potential and risk of sarcopenia: data from national health and nutrition examination surveys
Source: Aging (Albany NY). 2020 Dec 14;13(2):1913–28. doi: 10.18632/aging.202141 (PMC7880334; doi:10.18632/aging.202141)

## SUPPLEMENTARY FIGURE

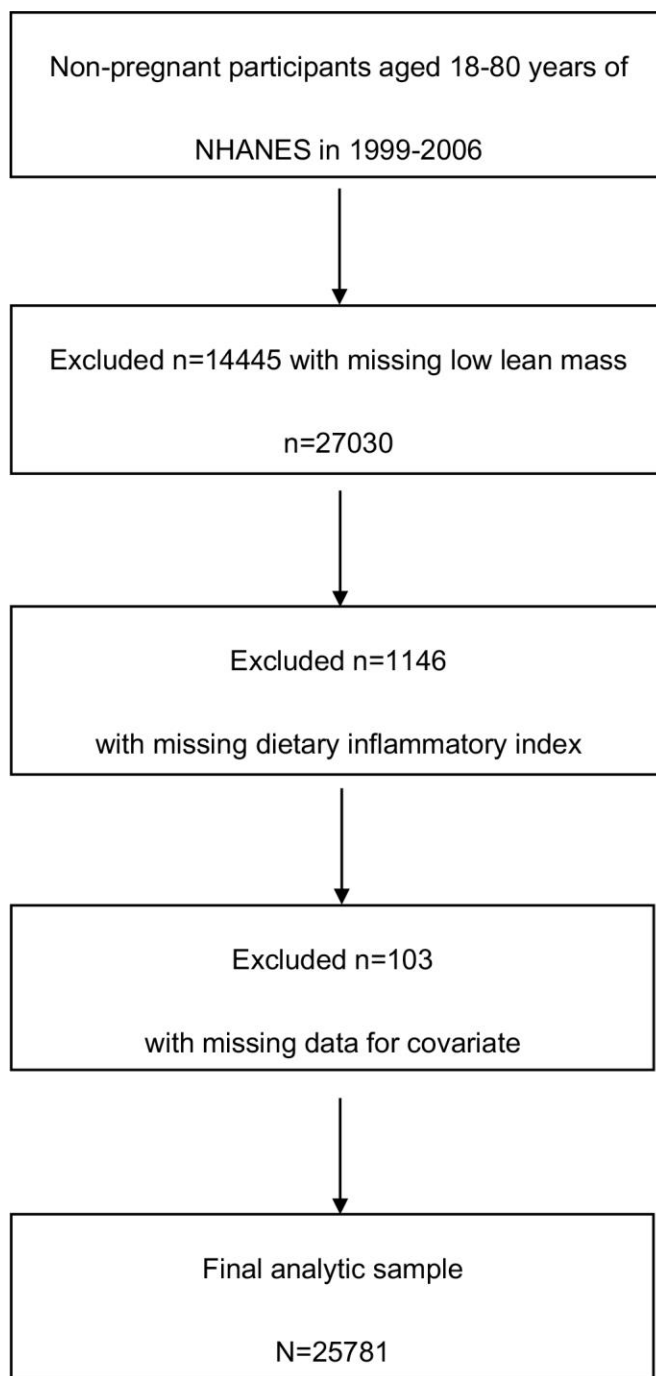

Supplementary Figure 1. NHANES 1999-2006 analytic sample flow chart.

Supplement: Supplementary Figure 1 [file aging-13-202141-s001.pdf]
