# Supplementary material for: Dietary inflammatory potential and risk of sarcopenia: data from national health and nutrition examination surveys
Source: Aging (Albany NY). 2020 Dec 14;13(2):1913–28. doi: 10.18632/aging.202141 (PMC7880334; doi:10.18632/aging.202141)
Supplement: Supplementary Tables [file aging-13-202141-s002.pdf]

SUPPLEMENTARY TABLES

Supplementary Table 1. Univariate analysis and multivariate analysis.

Exposure	β[1] (95% CI[2]), P value	
	Univariate	Multivariate
**Sex**		
**Male**	0.00(reference)	0.00(reference)
**Female**	0.60 (0.58, 0.63) <0.0001	0.73 (0.69, 0.78) <0.0001
**Age**	-0.02 (-0.02, -0.01) <0.0001	-0.00 (-0.00, -0.00) 0.0232
**Race**		
**Mexican American**	0.00(reference)	0.00(reference)
**Other Hispanic**	-0.09 (-0.14, -0.04) 0.0010	0.24 (0.15, 0.34) <0.0001
**Non-Hispanic White**	-0.15 (-0.19, -0.12) <0.0001	0.31 (0.25, 0.38) <0.0001
**Non-Hispanic Black**	0.23 (0.19, 0.27) <0.0001	0.54 (0.46, 0.61) <0.0001
**Other Race**	-0.30 (-0.36, -0.25) <0.0001	0.05 (-0.05, 0.15) 0.3314
**Ratio of family income to poverty**		
**<1.3**	0.00(reference)	0.00(reference)
**1.3-3.5**	-0.21 (-0.24, -0.18) <0.0001	-0.05 (-0.11, 0.00) 0.0562
**>3.5**	-0.67 (-0.71, -0.64) <0.0001	-0.33 (-0.39, -0.27) <0.0001
**Education level**		
**Less than high school**	0.00(reference)	0.00(reference)
**High school or GED General educational development**	-0.07 (-0.11, -0.03) 0.0002	-0.07 (-0.13, -0.01) 0.0316
**Above high school**	-0.45 (-0.48, -0.42) <0.0001	-0.33 (-0.39, -0.28) <0.0001
**Marital state**		
**Married or living with partner**	0.00(reference)	0.00(reference)
**Living alone**	0.16 (0.14, 0.19) <0.0001	0.06 (0.01, 0.10) 0.0170
**25BMI**		
**<25**	0.00(reference)	0.00(reference)
**≥25**	-0.29 (-0.32, -0.26) <0.0001	0.13 (0.08, 0.18) <0.0001
**Comorbidity index**		
**0**	0.00(reference)	0.00(reference)
**1**	0.22 (0.17, 0.26) <0.0001	0.07 (0.02, 0.12) 0.0075
**≥2**	0.33 (0.27, 0.39) <0.0001	0.12 (0.05, 0.20) 0.0014
**Smoking state**		
**Never**	0.00(reference)	0.00(reference)
**Former**	-0.12 (-0.16, -0.08) <0.0001	0.02 (-0.03, 0.07) 0.4656
**Current**	0.49 (0.43, 0.54) <0.0001	0.50 (0.44, 0.56) <0.0001
**Alcohol intake per week**		
**Never**	0.00(reference)	0.00(reference)
**Up to once a week**	-0.31 (-0.36, -0.26) <0.0001	-0.14 (-0.19, -0.08) <0.0001
**2-3 times a week**	-0.76 (-0.83, -0.69) <0.0001	-0.43 (-0.51, -0.36) <0.0001
**4-6 times a week**	-1.04 (-1.14, -0.95) <0.0001	-0.63 (-0.73, -0.54) <0.0001
**Daily or more**	-0.99 (-1.09, -0.89) <0.0001	-0.72 (-0.82, -0.61) <0.0001
**Physical activity**		
**Less than moderate**	0.00(reference)	0.00(reference)
**Moderate**	-0.15 (-0.19, -0.10) <0.0001	-0.11 (-0.17, -0.05) 0.0003
**Vigorous**	-0.37 (-0.41, -0.34) <0.0001	-0.39 (-0.44, -0.34) <0.0001

[1]β: effect sizes;
[2]95% CI: 95% Confidence interval.

Supplementary Table 2. Association of dietary inflammatory index with isokinetic strength of the knee extensors (quadriceps).

	Isokinetic Strength of the Knee Extensors (Quadriceps)		
**Dietary inflammatory index**	β[1] (95% CI[2]), P value		
	Model 1[3] (n=2983)	Model 2[4] (n=2086)	Model 3[5] (n=2016)
**Continuous**	-8.41 (-10.20, -6.62) <0.0001	-2.87 (-4.69, -1.06) 0.0020	-2.45 (-4.29, -0.61) 0.0090
**Tertiles**			
**Tertile 1(-5.18 to 1.20)**	1.00 (reference)	1.00 (reference)	1.00 (reference)
**Tertile 2(1.20 to 2.92)**	-22.45 (-30.73, -14.16) <0.0001	-10.67 (-18.53, -2.80) 0.0079	-10.56 (-18.50, -2.62) 0.0092
**Tertile 3(2.92 to 5.71)**	-36.64 (-45.17, -28.11) <0.0001	-11.62 (-20.12, -3.13) 0.0074	-9.21 (-17.89, -0.53) 0.0377
**DII group trend**	-9.71 (-11.92, -7.50) <0.0001	-3.32 (-5.51, -1.13) 0.0030	-2.79 (-5.02, -0.56) 0.0144

In sensitivity analysis, dietary inflammatory index was converted from a continuous variable to a categorical variable (tertiles).
[1]β: effect sizes;
[2]95% CI: 95% Confidence interval;
[3]Model 1: no covariates were adjusted;
[4]Model 2: adjusted for gender; age; race; ratio of family income to poverty; education level; BMI; comorbidity index; smoking; alcohol intake per week;
[5]Model 3: adjusted for gender; age; race; ratio of family income to poverty; education level; marital; BMI; comorbidity index; smoking; alcohol intake per week; physical activity.

Supplementary Table 3. Inflammatory effect scores.

Food parameter	Inflammatory effect score
Energy (kcal)	0.18
Alcohol (g)	-0.278
Cholesterol (mg)	0.11
Fat (g)	0.298
Fiber (g)	-0.663
Folic Acid (μg)	-0.19
β-carotene (μg)	-0.584
Iron (mg)	0.032
Magnesium (mg)	-0.484
Zinc (mg)	-0.313
Selenium (μg)	-0.191
Thiamin (mg)	-0.098
Vitamin A (RE)	-0.401
Vitamin B-6 (mg)	-0.365
Vitamin B-12 (μg)	0.106
Vitamin C (mg)	-0.424
Vitamin D (μg)	-0.446
Vitamin E (mg)	-0.419
Protein (g)	0.021
Niacin (mg)	-0.246
Riboflavin (mg)	-0.098
Carbohydrate (g)	0.097
Mono-unsaturated fatty acid	-0.009
Poly-unsaturated fatty acid	-0.337
Saturated fat	0.373
(n-3) Fatty acids	-0.436
(n-6) Fatty acids	-0.159
